# Prediction of low-addition segmented refractive intraocular lens position and deviation using anterior-segment optical coherence tomography

**DOI:** 10.1371/journal.pone.0305076

**Published:** 2024-06-10

**Authors:** Norihiro Mita, Mai Yamazaki, Yusuke Seki, Yu Sasaki, Eri Shibuya, Tsuyoshi Mito, Natsuko Hatsusaka, Eri Kubo, Hiroshi Sasaki

**Affiliations:** Department of Ophthalmology, Kanazawa Medical University, Kahoku, Ishikawa, Japan; University of Illinois at Chicago, UNITED STATES

## Abstract

This study aimed to develop and analyze the accuracy of predictive formulae for postoperative anterior chamber depth, tilt, and decentration of low-added-segment refractive intraocular lenses. This single-center, retrospective, observational study included the right eyes of 96 patients (mean age: 72.43 ± 6.58 years), who underwent a cataract surgery with implantation of a low-added segmented refractive intraocular lens at the Medical University Hospital between July 2019 and January 2021, and were followed up for more than 1 month postoperatively. The participants were divided into an estimation group to create a prediction formula and a validation group to verify the accuracy of the formula. Anterior segment optical coherence tomography (CASIA 2, Tomey Corporation, Japan) and swept-source optical coherence tomography biometry (IOL Master 700, Carl Zeiss Meditec AG) were used to measure the anterior ocular components. A predictive formula was devised for postoperative anterior chamber depth, intraocular lens tilt, and intraocular lens decentration (p <0.01) in the estimation group. A significant positive correlation was observed between the estimated values calculated using the prediction formula and the measured values for postoperative anterior chamber depth (r = 0.792), amount of intraocular lens tilt (r = 0.610), direction of intraocular lens tilt (r = 0.668), and amount of intraocular lens decentration (r = 0.431) (p < 0.01) in the validation group. In conclusion, our findings reveal that predicting the position of the low-added segmented refractive intraocular lens enables the prognosis of postoperative refractive values with a greater accuracy in determining the intraocular lens adaptation.

## Introduction

Harold Ridley [1] performed the first implantation of an intraocular lens (IOL) composed of polymethyl methacrylate in 1949. Since then, cataract surgery has evolved into a type of refractive surgery whose technique, IOL material, accuracy of preoperative examination, and structural design of the IOL have undergone considerable improvements and advancements [2–4]. Melles et al. [5] evaluated the postoperative refractive errors calculated using seven different types of IOL power calculation formulae and confirmed that approximately 80% of these errors were within ±0.5 D. Norrby [6] reported that postoperative refractive errors could be mainly attributed to errors in the predicted anterior chamber depth (ACD). Some IOL power calculation formulae based on ray tracing [7,8] have also been developed. Thus, the prediction of postoperative ACD contributes to improving the accuracy of IOL power calculations. Optical simulations [9–11] and clinical studies [12,13] have evaluated the influence of IOL tilt and decentration on visual function. Although several studies have reported on the postoperative ACD and preoperative prediction of the amount and direction of the tilt of the monofocal IOL [8,14–17], few studies have reported on the preoperative prediction of the amount and direction of IOL decentration.

Reportedly, Lentis Comfort (LS-313 MF15, Oculentis GmbH, Berlin, Germany; Santen) refractive IOLs with low addition (add) provide better visual acuity at distances ranging from 50 cm to 1 m, equivalent good contrast sensitivity compared to that by monofocal IOLs, and less postoperative spectacle dependence, with an incidence of subjective disturbing photic phenomena of less than 10% [18–20]. The design of the low-add segmented refractive IOL includes an optic measuring 6.0 mm in diameter and a plate haptic with an overall length of 11.0 mm. It is composed of an acrylate copolymer with an ultraviolet-absorbing hydrophobic surface having a refractive index of 1.46, haptic angulation of 0, and an IOL section of +1.5 D (spectacle plane: +1.06 D). The area ratio of the distance and intermediate zones for pupillary diameters exceeding 3 mm was approximately 3:2. In contrast, the proportion of the distance zone increased with a decrease in pupillary diameter [20]. Therefore, decentration has a substantial effect on the ratio of the distance and intermediate zones in the pupil, and greater decentration has a negative influence on the refractive values. Even a small decentration can have a large effect on postoperative refractive values, especially in eyes with small pupils. Thus, it is essential to predict the degree of decentration prior to cataract surgery. The IOL tilt negatively affects astigmatism and higher-order aberration. The decrease in the modulation transfer function (MTF) is influenced by the tilt of the low-add segmented refractive IOLs. Low-added segmented refractive IOLs have a greater coma aberration than that of monofocal IOLs; therefore, the tilt of low-add segmented refractive IOLs may further increase the coma aberration [21,22]. It is highly possible that the simultaneous occurrences of decentration and tilt have a substantial effect on postoperative visual function. Thus, the prediction of IOL eccentricity is extremely important for better planning and management of low-add segmented refractive IOL adaptations.

Therefore, this study aimed to devise methods for the prediction and verification of postoperative ACD and the amount and direction of the tilt and decentration of low-add segmented refractive IOLs.

## Material and methods

**Fig 1:**
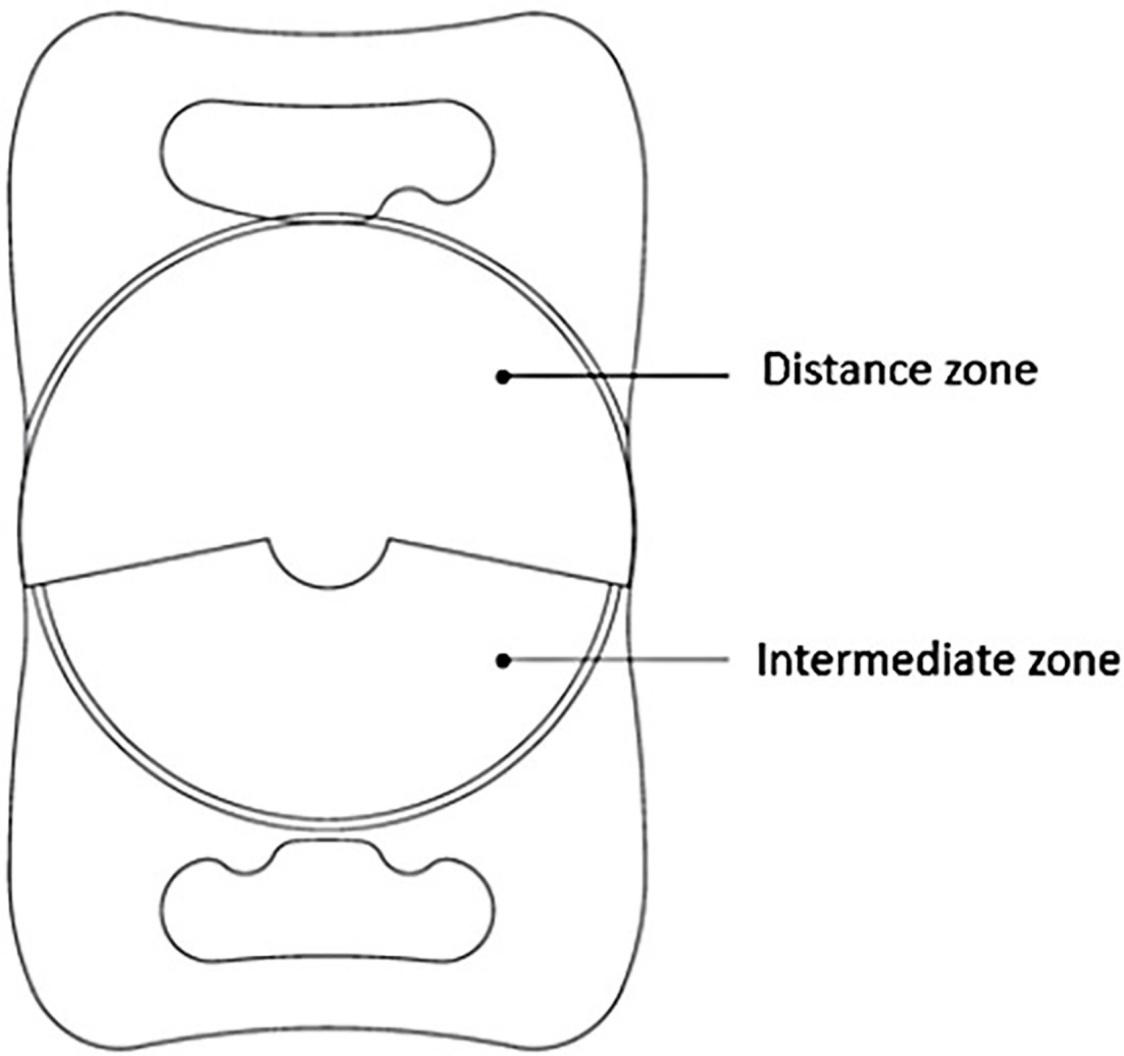
Low-added segmented refractive IOL (Lentis Comfort®, Santen).

This retrospective study included the right eyes of patients, who underwent a cataract surgery with implantation of a low-add segmented refractive IOL (Lentis Comfort [LS-313 MF15, Oculentis GmbH, Berlin, Germany; Santen]) at the Medical University Hospital between July 2019 and January 2021, and were followed up for more than 1 month postoperatively (Fig 1). Included patients had complete coverage with no cracks in the continuous curvilinear capsulorhexis; IOL insertion was in the bag. Furthermore, cases of zonular weakness, such as those associated with exfoliation syndrome, and corneal diseases, such as keratoconus, corneal dystrophy, and Fuchs Dystrophy, apart from refractive error, were excluded. This study was approved by the Ethics Review Committee of the Kanazawa Medical University (kahoku, Ishikawa, Japan) (approval no. H255) and adhered to the tenets of the Declaration of Helsinki.

From February 2021 to June 2021, access was granted to identifiable information of participants for the purpose of data collection. The analysis of the data was conducted after ensuring complete anonymization.

**Fig 2:**
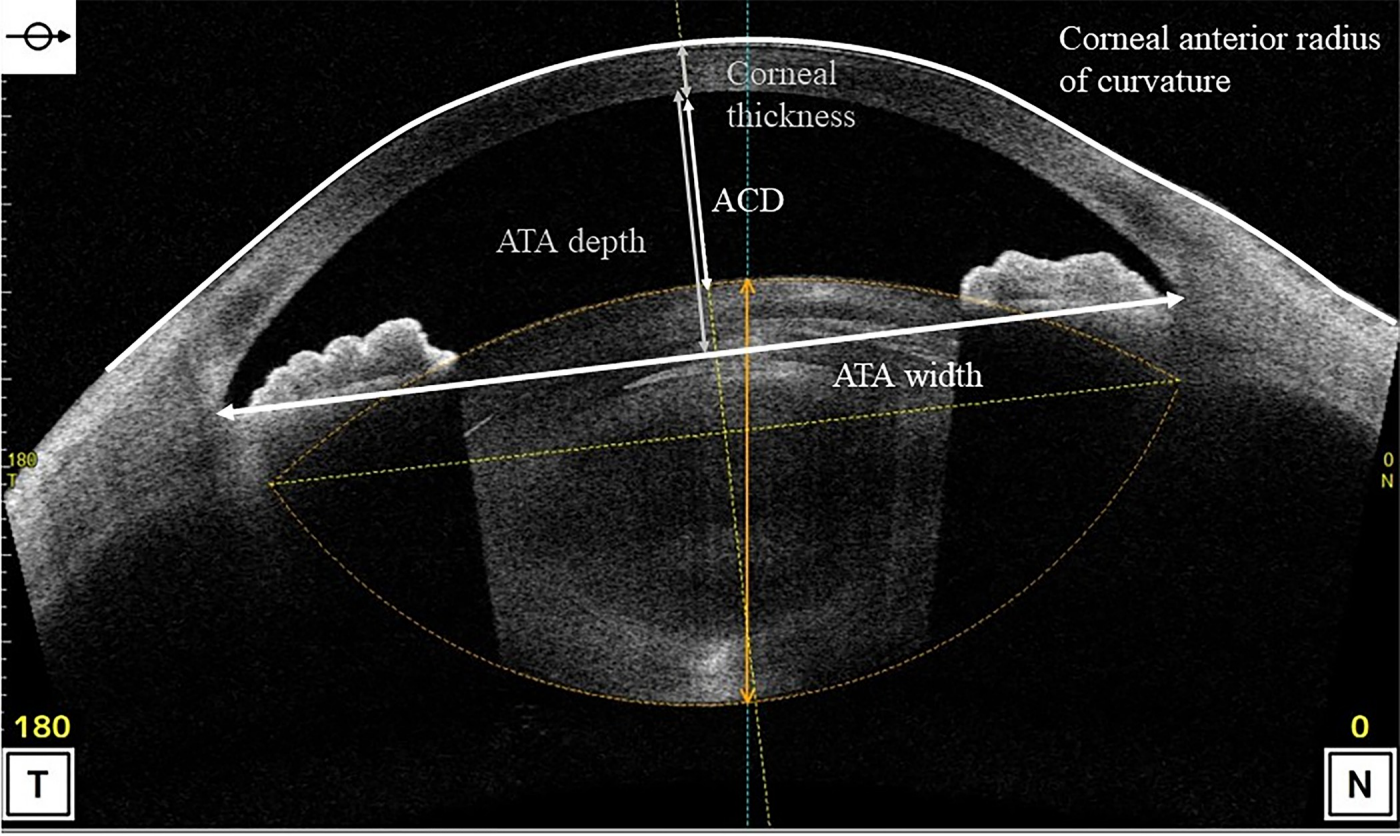
Preoperative analysis items for developing the prediction formula (from the corneal to the anterior surface of the crystalline lens).

**Fig 3:**
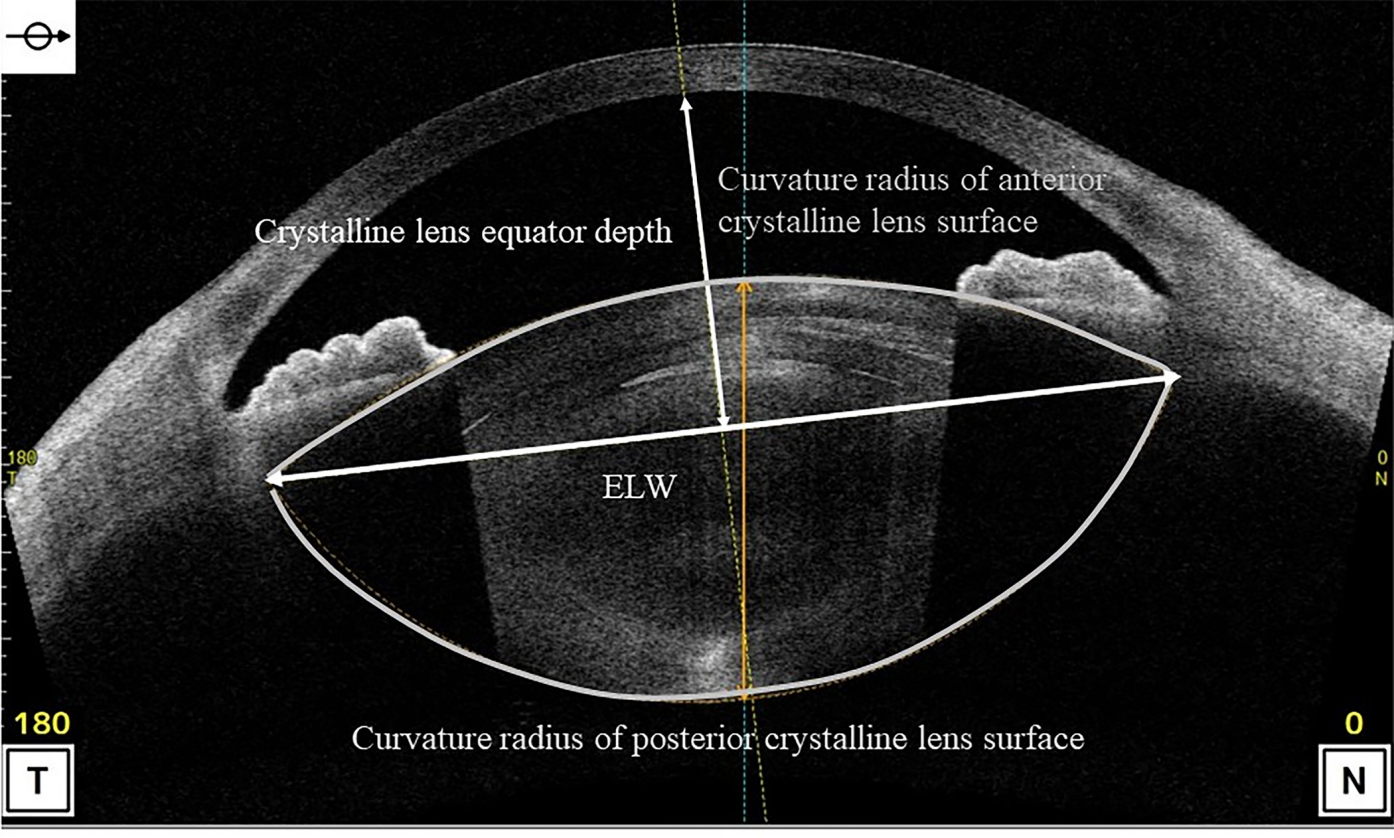
Preoperative analysis items for developing the prediction formula (the crystalline lens).

**Fig 4:**
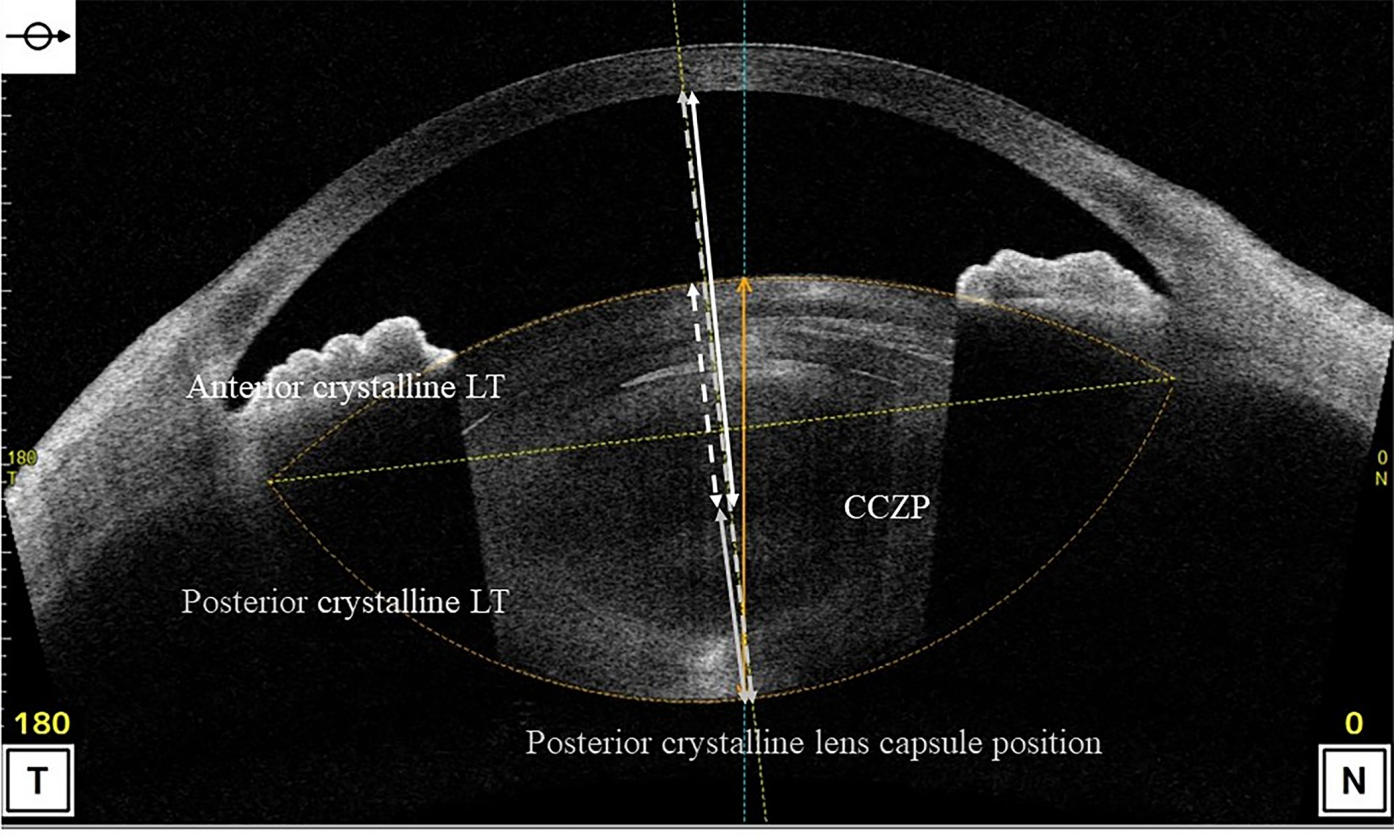
Preoperative analysis items for developing the prediction formula (the crystalline lens thickness and position).

**Fig 5:**
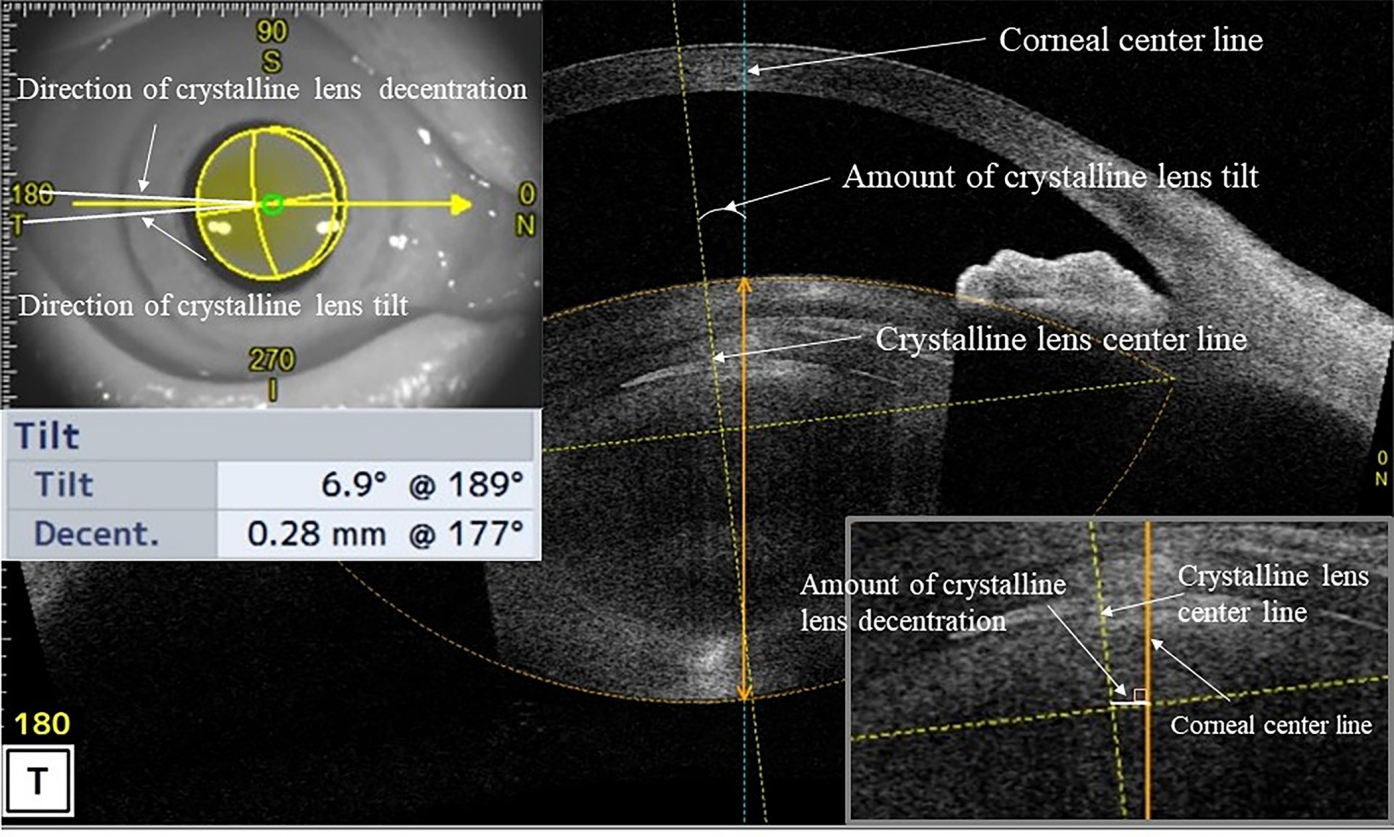
Direction and amount of crystalline lens in decentration and tilt.

**Fig 6:**
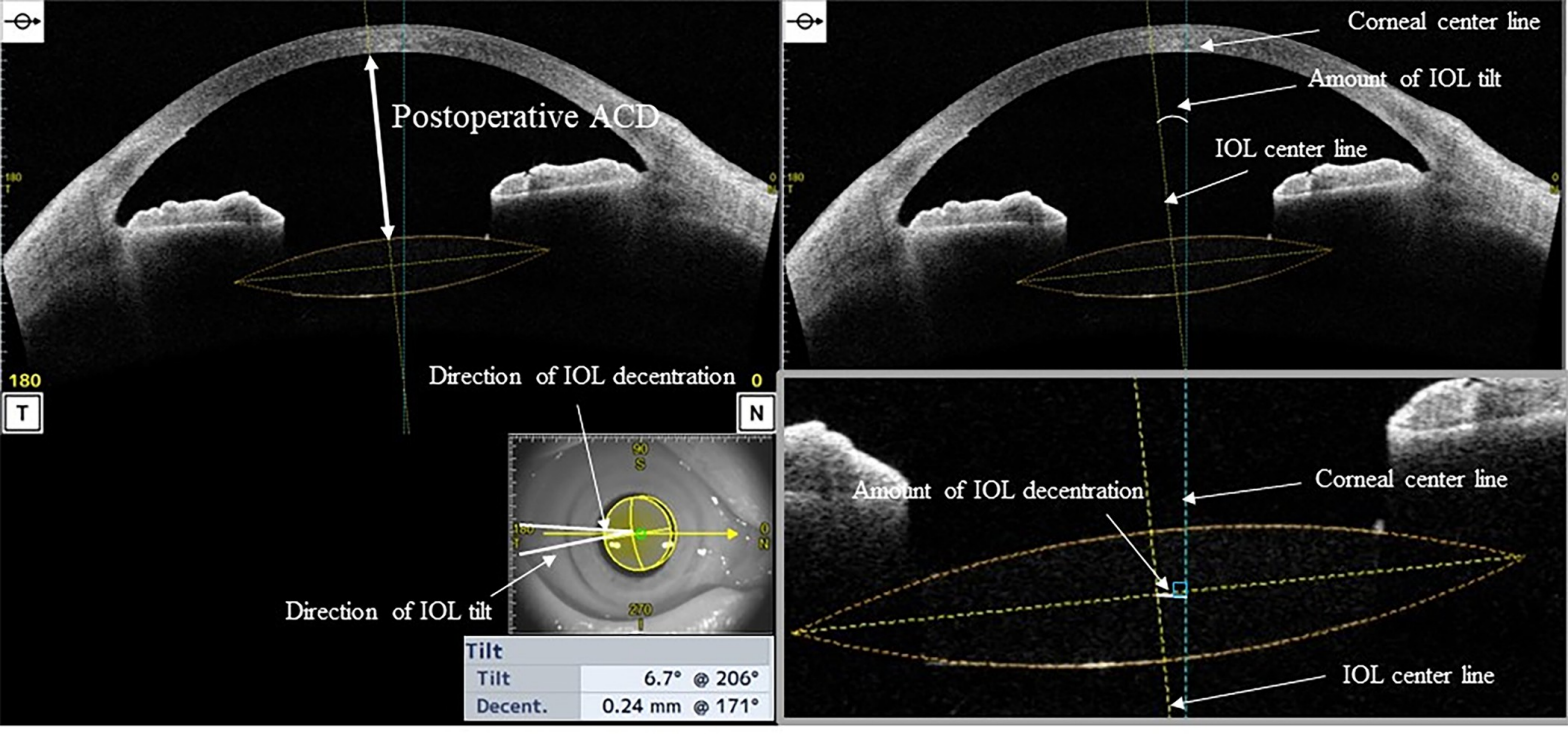
Postoperative analysis items for developing the prediction formula.

The anterior segment measurements were acquired using anterior segment optical coherence tomography (AS-OCT) (CASIA 2, Tomey Corporation,Nagoya,Japan) in a dark room under maximum mydriasis, which was achieved with topical instillation of phenylephrine hydrochloride and tropicamide drops before and 1 month after cataract surgery. The following preoperative parameters were measured in this study using a three-dimensional analysis: anterior radius of curvature of the cornea, corneal thickness, corneal diameter, ACD (from the posterior surface of the cornea (CP) to the anterior surface of the crystalline lens), angle-to-angle (ATA) depth (the perpendicular distance between CP and the intersection point of a line joining both the angle recesses with the corneal vertex), ATA width (Fig 2), lens equator depth [from the CP to the equatorial crystalline lens width (ELW)], central clear zone position (CCZP) (from the CP to the central clear zone), posterior crystalline lens capsule position (from the CP to the posterior crystalline lens capsule), crystalline lens thickness (LT), anterior LT (from the anterior surface to the central clear zone of the crystalline lens), posterior LT (the central clear zone of the crystalline lens to the posterior crystalline lens capsule), A/P ratio (anterior/posterior LT), ATA width and ELW, curvature radius of the anterior and posterior crystalline lens surface (Figs 3 and 4) and amount of crystalline lens tilt (i.e., the angle of intersection between the central cornea axis and central lens axis), direction of crystalline lens tilt (nasal: 0°, upper: 90°, temporal: 180°, and lower: 270°), amount of lens decentration (measured as the length of the perpendicular drawn from the central lens axis to the central corneal axis), and direction of crystalline lens decentration (nasal: 0°, upper: 90°, temporal: 180°, and lower: 270°) (Fig 5). The postoperative parameters included the ACD of IOL implanted eyes (from the CP to the anterior surface of the IOL), amount of IOL tilt (angle of intersection between the central cornea axis and central lens axis), direction of IOL (nasal: 0°, upper: 90°, temporal: 180°, and lower: 270°), amount of IOL decentration (length of the perpendicular from the central lens axis to the central corneal axis), and direction of IOL decentration (nasal: 0°, upper: 90°, temporal: 180°, and lower: 270°) (Fig 6). Axial length (AL) and corneal diameter were measured using swept-source OCT biometry (IOLMaster 700, Carl Zeiss Meditec AG). Easy R(EZR), which is available with R commander version 1.41, and includes the enhanced version of R and R commander (R Foundation for Statistical Computing, Vienna, Austria), was used for statistical analysis [23]. The forward stepwise selection was performed to develop a predictive formula for the IOL ACD, tilt, and decentration [14]. The Pearson product-moment correlation coefficient was used to evaluate the correlation between parameters at a significance level of 5%.

The participants were randomly divided into two groups: the estimation group, which was created to devise a prediction formula, and the validation group, which was created to verify the accuracy of the prediction formula.

## Results

In this study, the right eyes of 96 patients (mean age: 72.43 ± 6.58 years, sex: 37 males and 59 females) were included. The eyes were randomly assigned to the estimation (48 eyes) or validation (48 eyes) group. The characteristics of the estimation and validation groups are presented in Table 1. No significant differences were observed in age, sex, or any of the AS-OCT and IOL Master 700 parameters between the two groups. Both the groups were evaluated under identical conditions.

**Table 1:** Comparison between the estimation and validation groups.

Item	Estimation group	Validation group	p-value
Age (year):	71.9±6.5	73.0±6.6	0.432
Sex (male :1; female: 0)	0.396±0.494	0.375±0.489	0.836
Corneal anterior radius of curvature (mm)	44.050±1.106	44.223±1.780	0.569
Corneal thickness (mm)	0.533±0.034	0.516±0.034	0.074
Corneal diameter (mm)	11.835±0.347	11.809±0.414	0.747
ACD (mm)	2.822±0.381	2.815±0.344	0.924
Curvature radius of anterior crystalline lens surface (mm)	9.906±1.010	9.831±1.457	0.768
Curvature radius of posterior crystalline lens surface (mm)	5.681±0.604	5.594±0.562	0.469
Amount of crystalline lens tilt (mm)	4.713±1.113	4.421±1.432	0.268
Direction of crystalline lens tilt (°)	198.396±15.101	200.958±13.388	0.381
Amount of crystalline lens decentration (mm)	0.165±0.078	0.144±0.076	0.184
Direction of crystalline lens decentration (°)	186.333±49.382	182.729±75.815	0.783
ATA width (mm)	11.651±0.369	11.586±0.429	0.429
ELW (mm)	10.071±0.739	9.968±0.587	0.450
ATA depth (mm)	3.326±0.213	3.310±0.208	0.719
Crystalline lens equator depth (mm)	4.209±0.340	4.196±0.275	0.836
CCZP (mm)	5.245±0.307	5.242±0.281	0.970
Posterior crystalline lens capsule position (mm)	7.296±0.322	7.300±0.273	0.951
Crystalline LT (mm)	4.470±0.378	4.484±0.349	0.850
Anterior crystalline LT (mm)	2.419±0.261	2.427±0.233	0.872
Posterior crystalline lens thickness (mm)	2.051±0.163	2.057±0.191	0.869
Anterior lens thickness / Posterior lens thickness ratio	2.419±0.261	2.427±0.233	0.872
AL (mm)	24.031±1.139	23.994±1.392	0.887
Postoperative ACD (mm)	4.101±0.232	4.058±0.230	0.370
Amount of IOL tilt (mm)	4.640±1.184	4.417±1.272	0.376
Direction of IOL tilt (°)	204.146±12.810	205.729±16.502	0.601
Amount of IOL decentration (mm)	0.196±0.122	0.150±0.068	0.085
Direction of IOL decentration (°)	149.979±93.744	143.896±109.198	0.770

ACD, anterior chamber depth; ATA, angle-to-angle; ELW, equatorial crystalline lens width; CCZP, central clear zone position; LT, lens thickness; AL, axial length.

In the estimation group, multiple regression analysis (stepwise method) was used to predict the postoperative ACD, with CCZP, AL, ATA depth, and age as the best combinations (Table 2).

**Table 2:** Prediction formula for postoperative anterior chamber depth: a combination of explanatory variables.

Item	R^2^	p-value
CCZP	0.715	<0.001
CCZP, AL	0.743	<0.001
CCZP, AL, ATA depth	0.764	<0.001
CCZP, AL, ATA depth, Age	0.796	<0.001

CCZP, central clear zone position; AL, axial length; ATA, angle-to-angle.

**Fig 7:**
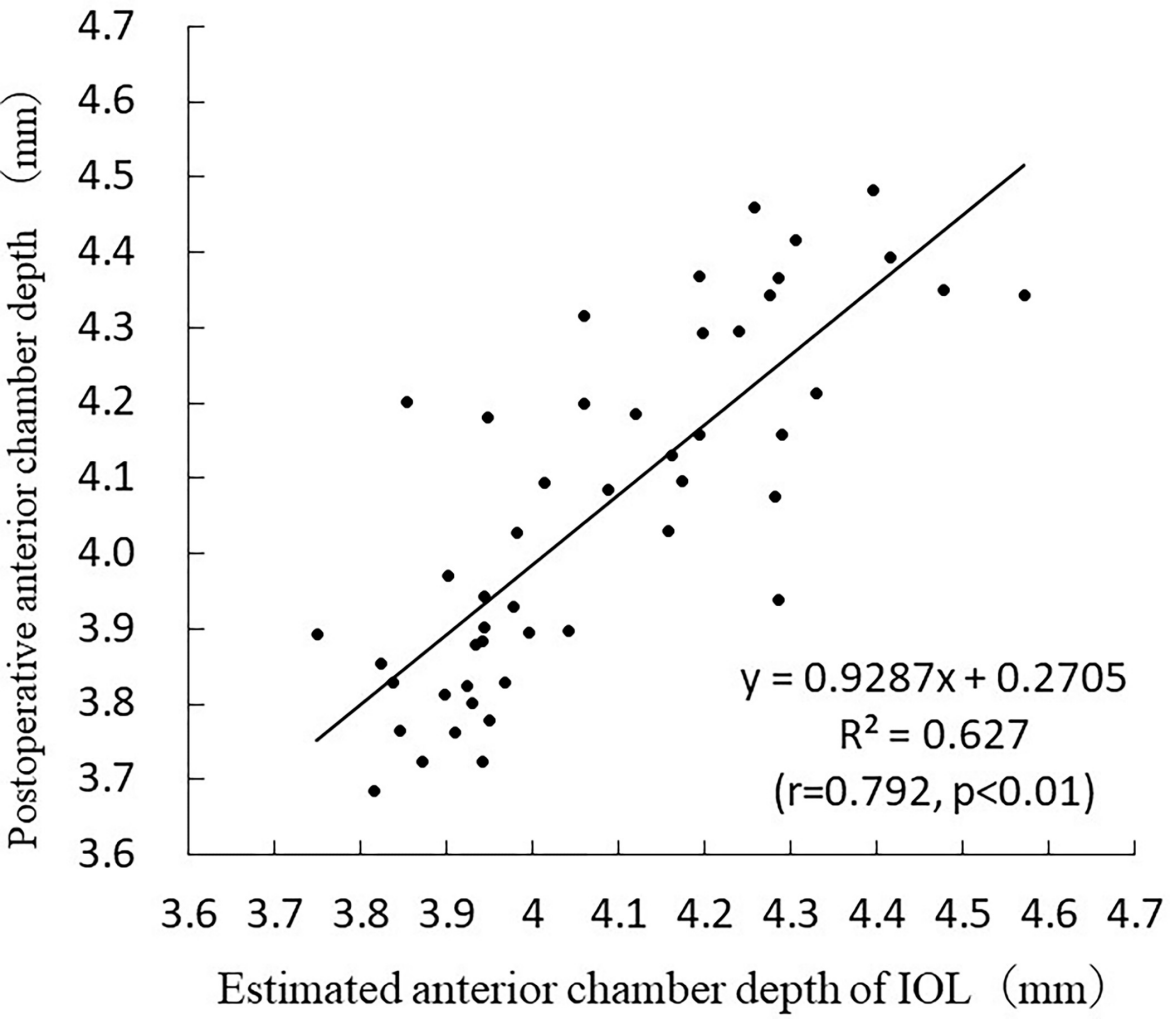
Correlation between estimated and actual values in postoperative anterior chamber depth.

The standard partial regression coefficients for the CCZP, AL, ATA depth, and age were 0.303, 0.304, 0.367, and 0.194, respectively. The following formula was developed based on the results of the stepwise analysis performed by the estimation group: Postoperative ACD = 0.580+0.062×AL+0.399 ×ATA depth+0.229 ×CCZP-0.007 ×age (R^2^ = 0.634, multicollinearity<3 in all categories; p < 0.001). The values obtained from the postoperative ACD formula were used as estimates for the validation group. The following simple linear regression analysis was performed based on the estimated and measured results in the validation group: Postoperative ACD = 0.928×estimated ACD of IOL+0.2705 (r = 0.792, p < 0.01) (Fig 7).

The postoperative ACD formulae were previously published by Goto [14], Sasaki [15], Young [16], Haigis [25], and Retzlaff (SRK/T) [26] with r values of 0.791, 0.780, 0.720, 0.672, and 0.584, respectively. The new postoperative ACD formula was better than the published results (Table 3).

**Table 3:** Correlation between estimated and measured postoperative anterior chamber depth in the comparison of new and previously published prediction formulae.

	Correlation between estimated and actual values in postoperative anterior chamber depth			
Study	Simple linear regression	R^2^	r	p-value
New prediction formula	y = 0.9287x + 0.2705	0.627	0.792	<0.01
Goto [14]	y = 0.8725x + 0.3828	0.626	0.791	<0.01
Sasaki [15]	y = 0.8716x + 0.4619	0.608	0.780	<0.01
Young [16]	y = 0.8510x - 0.1865	0.518	0.720	<0.01
Haigis [25]	y = 0.6801x + 1.0167	0.451	0.672	<0.01
Retzlaff(SRK/T) [26]	y = 0.3422x + 2.2000	0.341	0.584	<0.01

In the estimation group, multiple regression analysis (stepwise method) was used to predict the amount of postoperative IOL tilt, with the crystalline lens tilt, corneal thickness, and anterior crystalline LT being the best combinations (Table 4).

**Table 4:** Prediction formula for the amount of IOL tilt: a combination of explanatory variables.

Item	R^2^	p-value
Amount of crystalline lens tilt	0.647	<0.001
Amount of crystalline lens tilt, Corneal thickness	0.684	<0.001
Amount of crystalline lens tilt, Corneal thickness, Anterior crystalline LT	0.719	<0.001

LT: lens thickness.

**Fig 8:**
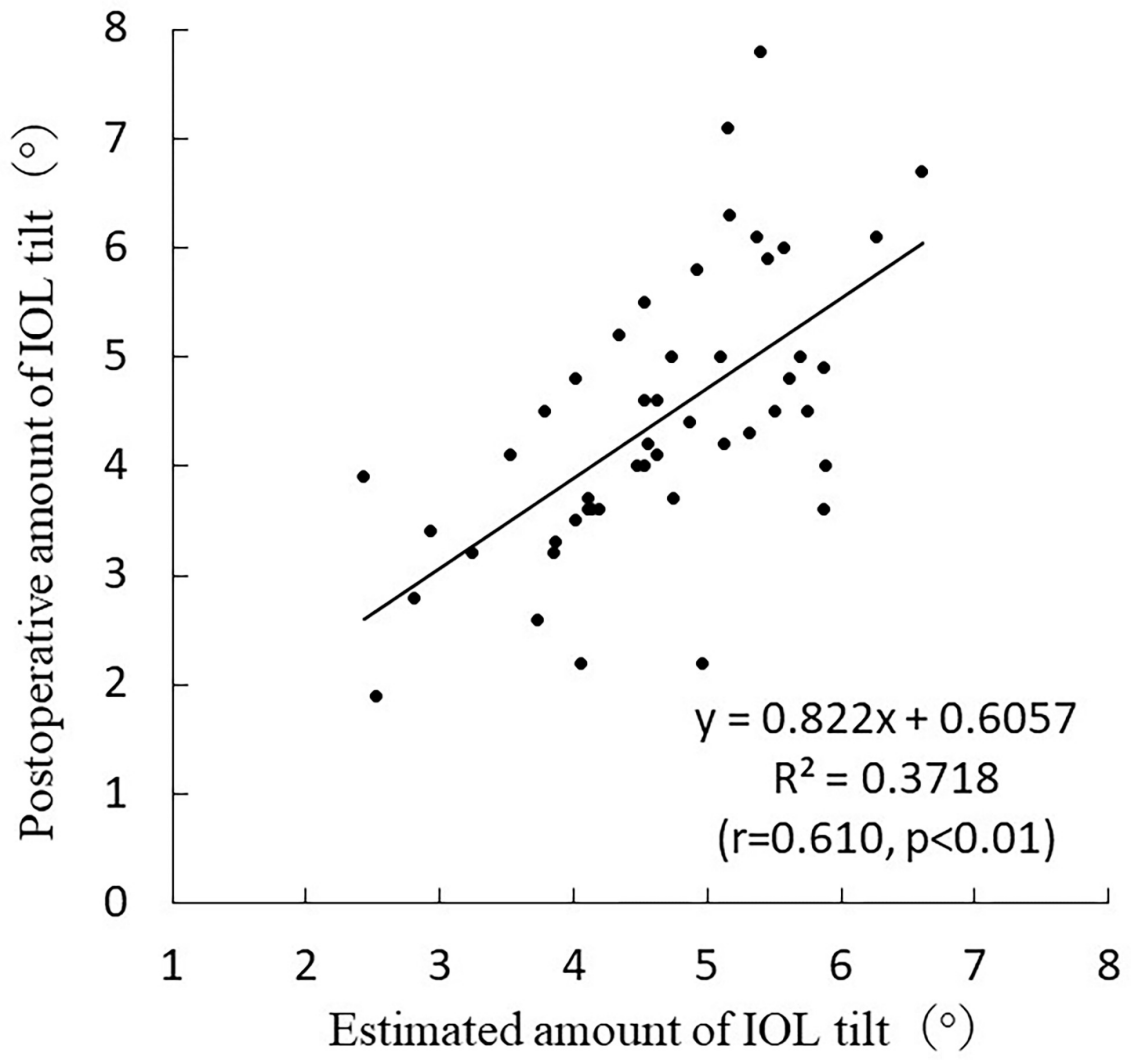
Correlation between estimated and actual values in postoperative amount of IOL tilt.

The standard partial regression coefficients for corneal thickness, crystalline lens tilt, and anterior crystalline LT were -0.295, 0.619, and 0.245, respectively. The following formula was developed based on the results of the stepwise analysis performed by the estimation group: Postoperative IOL tilt = 4.284–10.203×corneal thickness+0.659×amount of crystalline lens tilt+1.113 ×anterior crystalline LT (R^2^ = 0.517, multicollinearity <2 in all categories; p < 0.001). The values obtained from postoperative amount of IOL tilt formula were used as estimates for the validation group. The following simple linear regression analysis was performed based on the estimated and measured results in the validation group: Postoperative amount of IOL tilt = 0.822×estimated amount of IOL tilt+0.6057 (r = 0.610, p < 0.01) (Fig 8).

**Fig 9:**
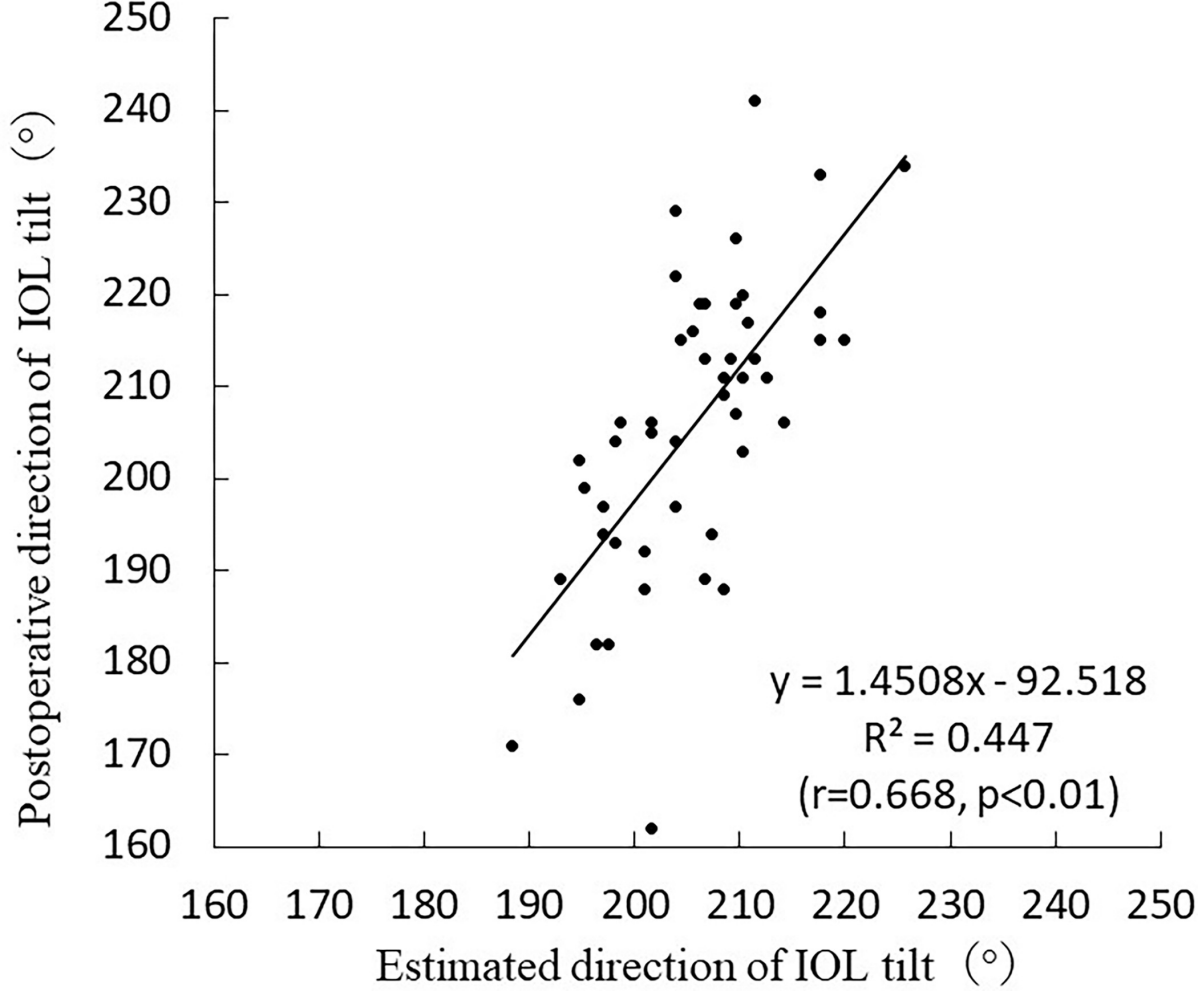
Correlation between the estimated and actual values in postoperative direction of IOL tilt.

In the estimation group, multiple regression analysis (stepwise method) was used to predict the postoperative direction of IOL tilt, with only the direction of the crystalline lens tilt being the best combination. The following formula was developed based on the results of the stepwise analysis performed by the estimation group: Postoperative direction of IOL tilt = 90.019+0.575 ×direction of crystalline lens tilt (R^2^ = 0.448, p < 0.001). The values obtained from the postoperative direction of the IOL tilt formula were used as estimates for the validation group. The following simple linear regression analysis was performed based on the estimated and measured results in the validation group: Postoperative direction of IOL tilt = 1.4508×estimated direction of IOL tilt-92.518 (r = 0.668, p < 0.01) (Fig 9).

In the estimation group, multiple regression analysis (stepwise method) was used to predict the postoperative amount of IOL decentration using the amount of crystalline lens decentration, with sex as the best combination (Table 5).

**Table 5:** Prediction formula for the amount of IOL decentration: a combination of explanatory variables.

Item	R^2^	p-value
Amount of crystalline lens decentration	0.282	<0.001
Amount of crystalline lens decentration Sex (male: 1 female: 0)	0.397	<0.001

The standard partial regression coefficients for the amount of crystalline lens decentration and sex were 0.341 and 0.309, respectively. The following formula was developed based on the results of the stepwise analysis performed by the estimation group: Postoperative estimated amount of IOL decentration = 0.077+0.539 ×amount of crystalline lens decentration+0.076×sex (R^2^ = 0.158, multicollinearity<2 in all categories; p < 0.001).

**Fig 10:**
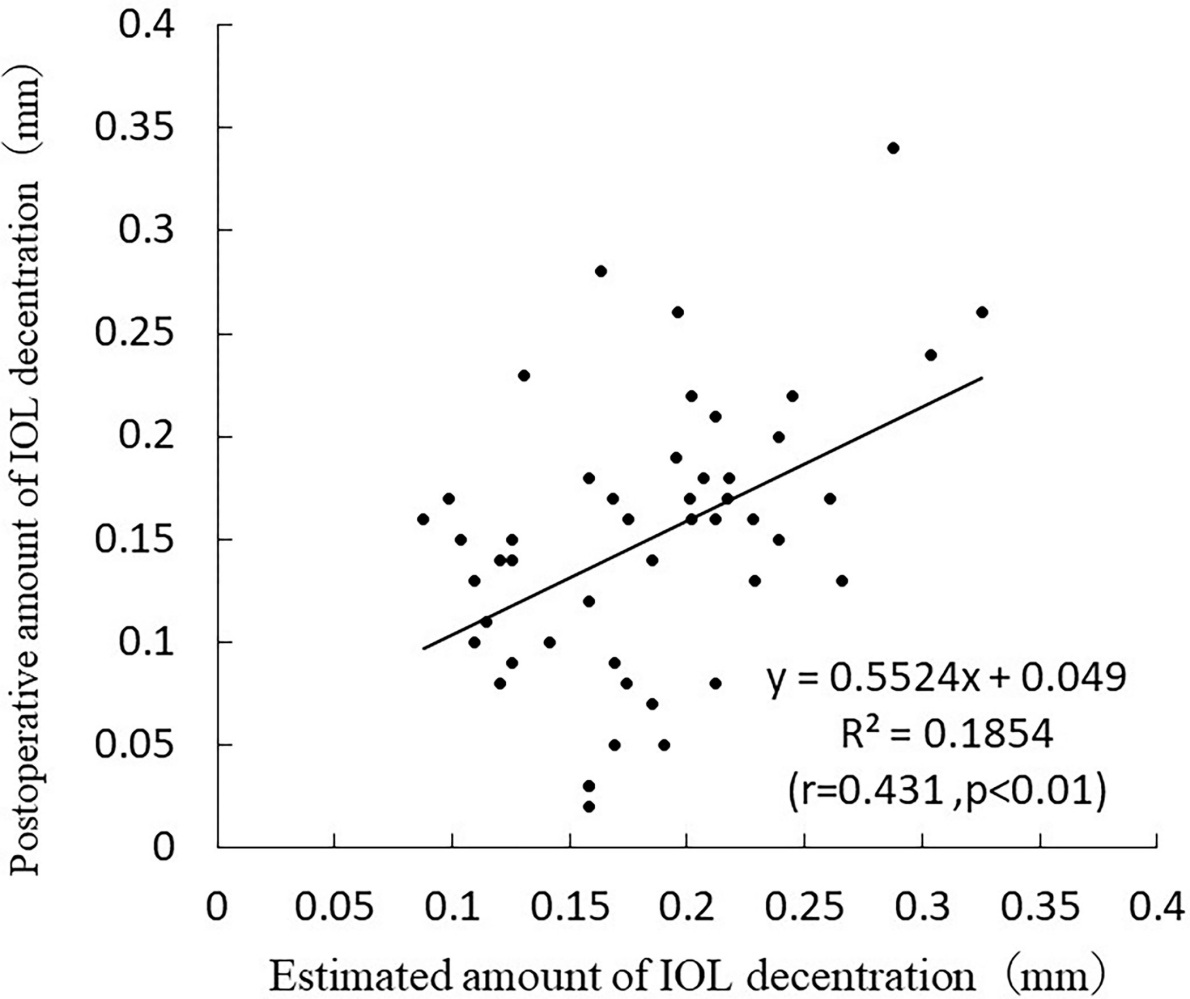
Correlation between the estimated and actual values in postoperative amount of IOL decentration.

The values obtained from postoperative amount of IOL decentration formula were used as estimates for the validation group. The following simple linear regression analysis is based on the estimated and measured results in the validation group. and postoperative amount of IOL decentration = 0.5524×estimated amount of IOL decentration+0.049 (r = 0.431, p < 0.01) (Fig 10).

In the estimation group, multiple regression analysis (stepwise method) was used to predict the postoperative direction of IOL decentration, with age, direction of crystalline lens tilt, and the ATA as the best combination (Table 6).

**Table 6:** Prediction formula for the direction of IOL decentration: A combination of explanatory variables.

Item	R^2^	p-value
Age	0.307	<0.001
Age, Direction of crystalline lens tilt	0.422	<0.001
Age, Direction of crystalline lens tilt, ATA depth	0.485	<0.001

ATA: angle-to-angle.

The standard partial regression coefficients for age, crystalline lens tilt direction, and ATA depth were 0.380, 0.311, and -0.266, respectively. The following formula was developed based on the results of the stepwise analysis performed by the estimation group. Postoperative direction of IOL decentration = -236.220+5.454×age+1.932×direction of crystalline lens decentration -117.062×ATA depth (R^2^ = 0.235, multicollinearity<2 in all categories; p < 0.001).

**Fig 11:**
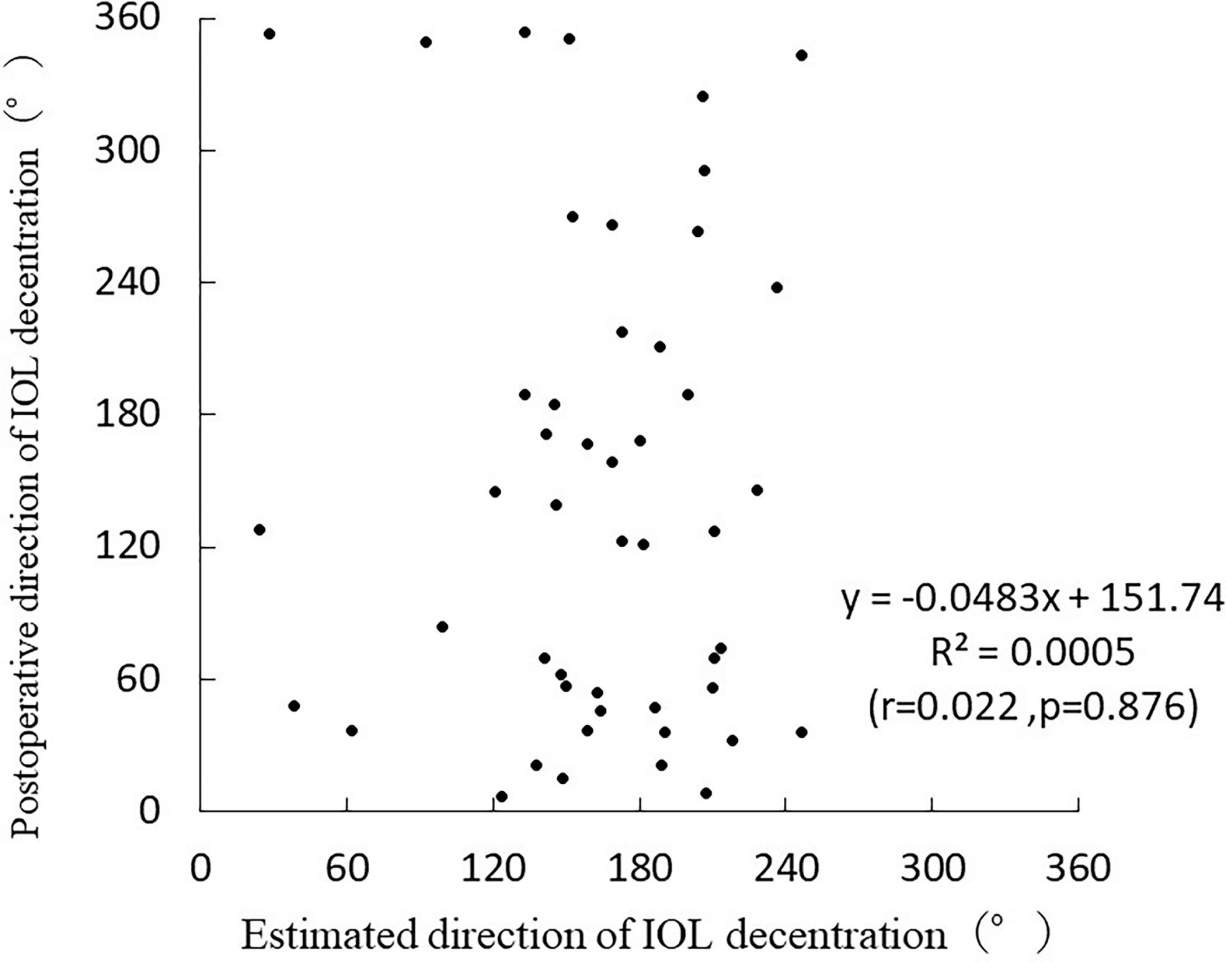
Correlation between the estimated and actual values in postoperative direction of IOL decentration.

The values obtained from the postoperative direction of the IOL decentration formula were used as estimates in the validation group. The following simple linear regression analysis was performed based on the estimated and measured results in the validation group: Postoperative direction of IOL decentration = -0.0483×estimated direction of IOL decentration+151.74 (r = 0.022, p = 0.876) (Fig 11).

No significant correlation was found between the estimated and actual values derived from the prediction formula for the postoperative direction of IOL decentration.

## Discussion

To the best of our knowledge, this is the first study to report the prediction of postoperative ACD and the amount and direction of IOL tilt and decentration, using preoperative parameters for low-added segmented refractive IOL. In this study of low-added segmented refractive IOLs, the prediction accuracy was found regarding preoperative parameters, including those measuring the anterior ocular structures, to be better for postoperative ACD, tilt, and decentration, in that order.

The measurement parameters used as predictors of postoperative ACD in this study were based on previous studies on monofocal IOLs [14–16], and additional factors related to the form of the ocular body were also considered. Postoperative ACD can be predicted with extremely high accuracy using preoperative parameters including AL, ATA depth, CCZP, and age. Goto et al. [14] reported that preoperative parameters such as ACD, AL, and ATA depth are extremely useful in predicting the postoperative ACD. Moreover, Sasaki et al. [15] and Yoo et al. [16] referred to CCZP and ELW, respectively, in their research.

Previous studies^14)-16), 25)26)^ have reported predictions using formulae for postoperative ACD, and the preoperative factors that comprise the prediction formula differ from each other.

Table 3 presents the correlation between the estimated values predicted by each of the previously reported prediction formulae and the actual values measured using the present data.

Although the type of IOL used in the previously reported prediction formulae was designed for a monofocal IOL, the new formulae were as accurate or more accurate than the previously reported formulae [14–16,24,25]. The AL and ATA, adopted as the prediction parameters in our formula, are stable factors that are not highly susceptible to changes before and after a cataract surgery. Moreover, the CCZP remained at approximately the same position irrespective of age [15], and the age factor comprehensively supplemented the other factors. In other words, our formula considered the positional relationship between anatomically stable factors and postoperative ACD. Consequently, the predictive accuracy was comparatively higher than that of previously reported prediction formulae [14–16,24,25]. The postoperative ACD affected the spherical values. Engren et al. [26] reported larger ACD-induced hypermetropia, even with the same implanted IOL, which was the principal factor responsible for postoperative refractive errors. Furthermore, Norrby [6] reported that the postoperative ACD error was the greatest factor responsible for postoperative refractive errors in IOL power calculations. Understanding the postoperative ACD from the perspective of preoperative parameters provides useful information for developing precise IOL power calculation formulae and facilitates the acquisition of better uncorrected visual acuity for emmetropia.

Vis-à-vis the postoperative amount and direction of IOL tilt, Hirnschall et al. [17] reported that the correlation between the amount of lens tilt and postoperative IOL tilt was stronger than that between the direction of preoperative crystalline lens tilt and postoperative IOL tilt. In contrast, our prediction formula yielded similar correlations for the amount of postoperative IOL tilt and direction of postoperative IOL tilt. The total higher-order and coma aberrations were higher in low-added segmented refractive IOLs compared with monofocal IOLs [21,22], which are described as a reduction in contrast sensitivity. As the postoperative IOL tilt increases the coma aberrations [27,28], it is necessary to predict the tilt and investigate the influence of coma aberrations caused by tilting in a low-added segmented refractive IOL.

The IOL tilt in this study ranged from 1.6 to 7.8°. The use of monofocal IOLs may be desirable if the expected tilt exceeds 5°, as Liu et al. [29] found in their optical simulation study that the MTF decreased at a tilt over 5°. Although the defocus curves of low-added segmented refractive IOLs indicated better visual acuity at distances ranging from 30 to 70 cm than that of monofocal IOLs and were equivalent to that of the extended-depth-of-focus IOL [30], the influence of decentration on visual acuity may be greater with low-added segmented refractive IOLs because of their vertical asymmetric design. The distance visual acuity is maintained even if the distance zone in the pupil increases owing to decentration, whereas the visual acuity at distances ranging from 30 to 70 cm decreases, and the entire distance visual acuity becomes equivalent to that of monofocal IOLs. If the intermediate zone increases due to decentration, the distance visual acuity may decrease due to a myopic shift. It is important to note that a slight decentration may have a substantial effect on the proportions of the distance and intermediate zones in the pupil.

Generally, the pupillary diameter is small in older individuals and tends to decrease with higher retinal illuminance [31]. The center of the low-added segmented refractive IOL was composed of a distance zone. The proportion of the distance zone was larger than that of the intermediate zone in eyes with a smaller pupillary diameter, which may reduce the visual acuity at distances of 30–70 cm. Therefore, the preoperative prediction of decentration in low-added segmented refractive IOLs could provide useful information for eyes with small pupillary diameters.

This study had some limitations. First, the degree of decentration was predicted based on preoperative measurements of the anterior ocular structures and other factors, whereas the direction of decentration could not be predicted because of the small sample size. Predicting the direction of IOL decentration would have enabled a more detailed prognosis of other occurrences, such as the effect on visual acuity at different distances. We believe that it would be possible to create a formula for predicting the direction of decentration with higher accuracy in each direction of plate insertion, if future studies incorporate a larger sample population. Second, although the prediction of the postoperative IOL position was possible, the development of an IOL power calculation formula that incorporates ACD remains a challenge, and it could not be surmounted in this study. Third, the κ angle [32] was not considered in this study. The κ angle affects higher-order aberrations [33] and photopic phenomena [34], and may reduce higher-order aberrations caused by tilting or decentration of the IOL. The postoperative prediction of the κ angle should be performed using the same procedure as that for the prediction of the postoperative IOL position.

Finally, the predictive formula we have developed does not consider complications other than cataracts. Exfoliation Syndrome [35] changes in the position and decentration of the lens are expected, which may reduce the accuracy of postoperative IOL position and decentration predictions. Furthermore, corneal diseases such as keratoconus [36], corneal dystrophy [37], and Fuchs Dystrophy [38] affect the corneal anterior radius of our predictive formula of curvature, corneal thickness, ATA depth, central clear zone position (CCZP), and axial length (AL), thus reducing the prediction accuracy of IOL position and tilt. Anterior capsule contraction [39] varies with lens shape and material, hence incorporating these factors could improve the prediction accuracy.

In conclusion, predicting the position of the low-added segmented refractive IOL enables the creation of a prediction formula for ACD, the amount and direction of tilt, and the amount of decentration. Furthermore, it facilitates the prognosis of postoperative refractive values with a greater accuracy in determining the adaptation of low-added segmented refractive IOLs.
